# Serotonergic Modulation of Persistent Inward Currents in Serotonergic Neurons of Medulla in ePet-EYFP Mice

**DOI:** 10.3389/fncir.2021.657445

**Published:** 2021-04-06

**Authors:** Yi Cheng, Nan Song, Renkai Ge, Yue Dai

**Affiliations:** ^1^School of Physical Education, Yunnan University, Kunming, China; ^2^Key Laboratory of Adolescent Health Assessment and Exercise Intervention of Ministry of Education, School of Physical Education and Health Care, East China Normal University, Shanghai, China; ^3^Shanghai Key Laboratory of Multidimensional Information Processing, School of Communication and Electronic Engineering, East China Normal University, Shanghai, China; ^4^School of Physical Education and Health Care, East China Jiaotong University, Nanchang, China

**Keywords:** serotonergic neurons, neuromodulation, persistent inward currents, neuronal excitability, medulla

## Abstract

Serotonergic (5-HT) neurons in the medulla play multiple functional roles associated with many symptoms and motor activities. The descending serotonergic pathway from medulla is essential for initiating locomotion. However, the ionic properties of 5-HT neurons in the medulla remain unclear. Using whole-cell patch-clamp technique, we studied the biophysical and modulatory properties of persistent inward currents (PICs) in 5-HT neurons of medulla in ePet-EYFP transgenic mice (P3–P6). PICs were recorded by a family of voltage bi-ramps (10-s duration, 40-mV peak step), and the ascending and descending PICs were mirrored to analyze the PIC hysteresis. PICs were found in 77% of 5-HT neurons (198/258) with no significant difference between parapyramidal region (*n* = 107) and midline raphe nuclei (MRN) (*n* = 91) in either PIC onset (−47.4 ± 10 mV and −48.7 ± 7 mV; *P* = 0.44) or PIC amplitude (226.9 ± 138 pA and 259.2 ± 141 pA; *P* = 0.29). Ninety-six percentage (191/198) of the 5-HT neurons displayed counterclockwise hysteresis and four percentage (7/198) exhibited the clockwise hysteresis. The composite PICs could be differentiated as calcium component (Ca_PIC) by bath application of nimodipine (25 μM), sodium component (Na_PIC) by tetrodotoxin (TTX, 2 μM), and TTX- and dihydropyridine-resistance component (TDR_PIC) by TTX and nimodipine. Ca_PIC, Na_PIC and TDR_PIC all contributed to upregulation of excitability of 5-HT neurons. 5-HT (15 μM) enhanced the PICs, including a 26% increase in amplitude of the compound currents of Ca_PIC and TDR_PIC (*P* < 0.001, *n* = 9), 3.6 ± 5 mV hyperpolarization of Na_PIC and TDR_PIC onset (*P* < 0.05, *n* = 12), 30% increase in amplitude of TDR_PIC (*P* < 0.01), and 2.0 ± 3 mV hyperpolarization of TDR_PIC onset (*P* < 0.05, *n* = 18). 5-HT also facilitated repetitive firing of 5-HT neurons through modulation of composite PIC, Na_PIC and TDR_PIC, and Ca_PIC and TDR_PIC, respectively. In particular, the high voltage-activated TDR_PIC facilitated the repetitive firing in higher membrane potential, and this facilitation could be amplified by 5-HT. Morphological data analysis indicated that the dendrites of 5-HT neurons possessed dense spherical varicosities intensively crossing 5-HT neurons in medulla. We characterized the PICs in 5-HT neurons and unveiled the mechanism underlying upregulation of excitability of 5-HT neurons through serotonergic modulation of PICs. This study provided insight into channel mechanisms responsible for the serotonergic modulation of serotonergic neurons in brainstem.

## Introduction

Serotonergic (5-HT) neurons in the medulla play multiple functional roles and are associated with symptoms such as depression (Meera et al., 2003) and pain (Potrebic et al., 1994), or behaviors like locomotion (Liu and Jordan, 2005), respiration (Severson et al., 2003), and perception (Geyer and Vollenweider, 2008). The descending serotonergic pathway originating from medulla plays an important role in initiating and controlling the rhythmic motor movement such as locomotion (Noga et al., 2017). Previous study reported that electrical stimulation of 5-HT neurons of the parapyramidal region (PPR) in medulla could initiate locomotion in isolated rat spinal cord (Liu and Jordan, 2005). Results from previous studies also showed that bath application of 5-HT elicited locomotor-like activity in the isolated spinal cord preparation (Cowley and Schmidt, 1994; Kjaerulff and Kiehn, 1996). 5-HT neurons in medulla have been characterized in a recent study and the data showed that the 5-HT neurons could be classified into parapyramidal region (PPR) and midline raphe nuclei (MRN) neurons with no significant difference in membrane properties between the PPR and MRN (Dai et al., 2016). However, the ionic properties of the 5-HT neurons, especially the persistent inward currents (PICs) which regulate the neuronal excitabilities have not been well-studied, yet.

Persistent inward currents (PICs) are voltage-dependent currents that have been found in many types of neurons in vertebrates (Perrier and Tresch, 2005; Zhong et al., 2007). In spinal motoneurones, PICs play an essential role in regulating neuronal excitability and motor output (Heckman et al., 2008; Dai et al., 2018). PICs in spinal neurons are generally composed of sodium and calcium currents (Dai and Jordan, 2010). The sodium component of PIC (Na_PIC) is tetrodotoxin (TTX) sensitive while the calcium component of PIC (Ca_PIC) is dihydropyridine (DHP) sensitive (Hounsgaard and Mintz, 1988). A novel PIC which is TTX and DHP resistant (TDR_PIC) was reported in neonatal mouse spinal neurons (Dai and Jordan, 2011) and brainstem 5-HT neurons (Dai et al., 2016; Dai and Cheng, 2019; Cheng et al., 2020) and was shown to be mediated by sodium currents in spinal neurons. Although PICs have been studied intensively in many types of neurons, the biophysical parameters and modulatory properties of PICs in medullar 5-HT neurons are still missing.

Previous studies reported that serotonergic axons from the dorsal raphe (DR) nucleus are very fine and typically have small, pleomorphic varicosities. These fibers branch profusely in their vicinity areas and diffuse 5-HT through small varicosities (Colgan et al., 2012; Quentin et al., 2018). Such innervations have considerable physiological and/or pharmacological importance as 5-HT released in the vicinity of serotonergic cell bodies regulates the firing of serotonergic neurons through activation of somatodendritic autoreceptors (Jennings, 2013). In a recent study, we discovered large amounts of small varicosities in the dendrites of 5-HT neurons in medulla. However, it remains unknown about the implication of morphological characteristics for potential serotonergic modulation of 5-HT neurons.

Using ePet-EYFP mice, in this study we characterized PICs in 5-HT neurons of medulla with electrophysiological and pharmacological properties. We also explored functional role of the PICs in regulating neuronal excitability as well as serotonergic modulation of the PICs in 5-HT neurons. Our data showed that 5-HT enhanced PICs in medullar 5-HT neurons in ePet-EYFP mice. Preliminary data was published in abstract form (Cheng et al., 2019b; Dai and Cheng, 2019).

## Materials and Methods

### Animal Model

Experiments were performed in accordance with the East China Normal University Laboratory Animal Center and all procedures were in accordance with protocols approved by the Animal Experiment Ethics Committee (Ethics No. m20190201). The experiments were carried out on neonatal ePet-EYFP mice (P3–P6), crossed by ePet-cre mice (The Jackson Laboratory, stock no. 012712) with R26-stop-EYFP mice (The Jackson Laboratory, stock no. 006148). Animals were exposed to a 12 h light/dark cycle and had free access to food and water. Their pain and distress were minimized.

### Preparation of Slices and Patch-Clamp Recordings

**FIGURE 1:**
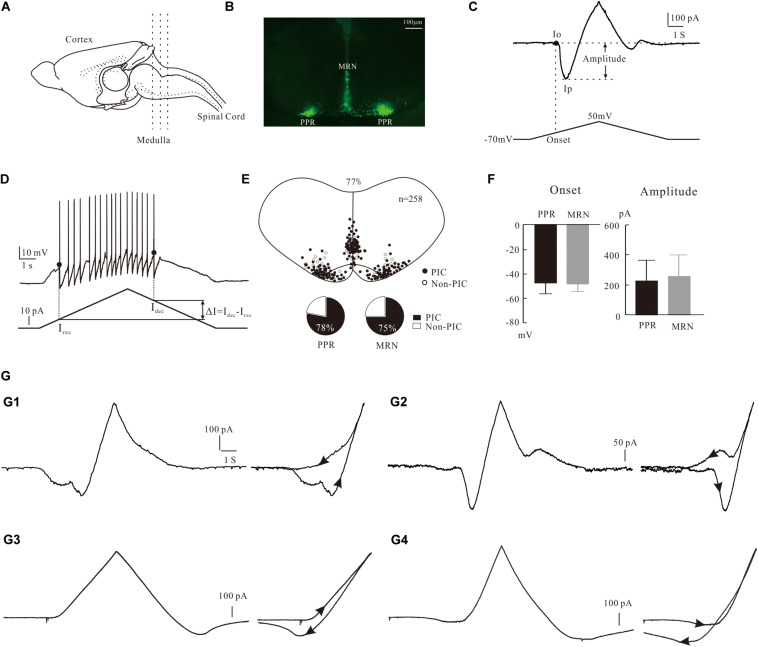
Characterization of PICs in 5-HT neurons. **(A)** The transverse slices were collected from the medulla indicated by dash lines. **(B)** One slice cut from the sections shows the EYFP^+^ serotonin (5-HT) neurons with enhanced yellow fluorescent proteins in parapyramidal region (PPR) and midline raphe nuclei (MRN) areas. **(C)** Measurement of biophysical parameters of PICs by the voltage bi-ramp from −70 to 50 mV. **(D)** Measurement of biophysical parameters (recruitment currents, decruitment currents, and difference ΔI) from repetitive firing induced by current bi-ramp with a duration of 10 s, peak of 60–80 pA, and holding current of zero pA. **(E)** Distribution of 5-HT neurons with PICs in medulla (top). Proportions of PICs (solid) and Non-PICs (open) recorded from PPR and MRN 5-HT neurons (bottom). **(F)** Statistic results of the onset and amplitude of PICs in PPR (*n* = 107) and MRN (*n* = 91). Error bars show SD, unpaired *t*-test. **(G)** Four patterns of PICs in 5-HT neurons of medulla. **(G1)** a-PIC only with counterclockwise hysteresis. **(G2)** a-PIC > d-PIC with counterclockwise hysteresis of PICs. **(G3)** d-PIC only with clockwise hysteresis. **(G4)** d-PIC > a-PIC with clockwise hysteresis of PICs.

The general experimental and surgical procedures have been described in details in previous study (Cheng et al., 2020). Briefly, the postnatal day 3–6 ePet-EYFP mice of either sex were euthanized by cervical dislocation and quickly decapitated. A section of medulla was removed and glued to a Plexiglas tray filled with cooled dissecting artificial cerebrospinal fluid (ACSF), bubbled with 95% O_2_ and 5% CO_2_. Three transverse slices 200 μm thick were cut from the ponto-medullary junction (Figure 1A), transferred to a holding chamber, and incubated at room temperature (20–22°C) for 60 min recover in recording ACSF.

Slices were then transferred to a recording chamber mounted in the stage of an upright Olympus BX50 microscope fitted with differential interference contrast (DIC) optics and epifluorescence. The chamber was perfused with recording ACSF at rate of 2 ml/min, bubbled with 95% O_2_ and 5% CO_2_. The EYFP^+^ 5-HT neurons were identified at X40 magnification using epifluorescence with a narrow band YFP cube (Figure 1B). The visualized EYFP^+^ neurons were patched with glass pipette electrodes. The pipette electrodes were pulled from borosilicate glass (1B150F-4; WPI) with an electrode puller (P-1000; Sutter Instrument) and had resistances of 6–8 MΩ when filled with intracellular solution. A MultiClamp 700B, a Digidata 1550, a MiniDigi 1B, and pCLAMP 10.7 (all from Molecular Devices) were used for data acquisition. Data were low-pass filtered at 3 kHz and sampled at 10 kHz. Whole cell patch recordings were made in voltage-clamp mode with 85% capacitance compensation and current-clamp mode with bridge balance. Electrophysiological data were analyzed with Axon Clampfit (10.7). Data are presented as means ± SD. Unpaired *t*-test (Graphpad Prism 8) was performed to compare the PIC parameters 5-HT neurons in PPR and MRN. Paired *t*-test (Graphpad Prism 8) was used to analyze the effect of blockers on PIC parameters and the effect of 5-HT on neuronal membrane properties. *P* < 0.05 was for significant tests.

### Measurement of PICs Parameters

PICs were recorded by applying a family of five slow voltage bi-ramps (10 s duration, 40 mV step of peak) to the neurons. Normally the best of recordings from the same steps from both control and conditions were chosen to calculate the PICs parameters. Details of the measurement are shown in Figure 1C, where the leak current was subtracted in all neurons before calculating the amplitude and onset of the PICs. A straight line (dashed line) was drawn along the rising phase of the current trace. The last point where the straight line was tangent to the current trace was defined as the onset of PIC (I_o_), and the corresponding voltage on the voltage ramp was defined as the onset voltage of PIC. The lowest point on the current trough was defined as the peak of PIC (I_p_). The amplitude PIC was calculated as the difference between I_o_ and I_p_, i.e., PIC = I_o_ – I_p_. Details of measurement of PIC were described in previous studies (Dai and Jordan, 2010). The current trace between Io and Ip was fitted by the Boltzmann equation f(V) = 1/{1 + exp[(Vmid – V)/Vc]} for determination of kinetics of PIC (V_mid_ and V_c_).

In order to investigate the contribution of PICs to the regulation of neuronal firing properties, we recorded 5-HT neurons in current clamp mode, where a slow current bi-ramp with a duration of 10 s, peak of 60–80 pA, and holding current of zero pA was applied to the neurons. The instantaneous frequency of firing was calculated. The recruitment current (I_rec_) was defined as the point of the depolarizing current ramp at which the first spike was initiated, and the decruitment current (I_dec_) as the point of the repolarizing current ramp at which the last spike was generated. And then we calculated the difference ΔI = I_dec_ – I_rec_ (Figure 1D). The voltage threshold (*V*th) was defined as the membrane potential at which the rising rate d*V*/d*t* ≥ 10 mV/ms. The parameters measured and calculated in this study included the resting membrane potential (RMP), voltage threshold (Vth), current threshold (rheobase), input resistance (Rin), action potential (AP) height and half-width, afterhyperpolarization (AHP) depth, and half-decay time. The definition and calculation of these parameters were described in details in our previous study (Cheng et al., 2019a). To unify standards, the AP parameters were calculated from the first spike of firings evoked by current bi-ramp. Cells selected for data analysis must meet the following conditions: stable resting membrane potential between −55 and −70 mV, input resistance ≥ 300 MΩ, action potential amplitude ≥ 40 mV, and time for intracellular recording ≥ 20 min.

### Images of Labeling Cells

Some cells were labeled with 3% tetramethylrhodamine in the recording pipettes. The photos of labeled neurons were taken immediately by a Nikon Eclipse Ni fluorescence microscopy with a Nikon DS-Ri2 color digital camera at 540–580 nm and 465–495 nm excitation wavelengths, separately.

### Solutions and Chemicals

Dissecting ACSF (μM): NaCl (25), sucrose (253), KCl (1.9), NaH_2_PO_4_ (1.2), MgSO_4_ (10), NaHCO_3_ (26), kynurenic acid (1.5), glucose (25), and CaCl_2_ (1.0).

Recording ACSF (μM) for voltage clamp: NaCl (125), KCl (2.5), NaHCO_3_ (26), NaH_2_PO_4_ (1.25), glucose (25), MgCl_2_ (1), Cl-TEA (10), and CaCl_2_ (2.0).

Recording ACSF (μM) for current clamp: NaCl (125), KCl (2.5), NaHCO_3_ (26), NaH_2_PO_4_ (1.25), glucose (25), MgCl_2_ (1), and CaCl_2_ (2.0).

Intracellular solution (μM) for voltage clamp: K-gluconate (135), NaCl (10), Cl-TEA (20), HEPES (10), MgCl_2_ (2), Mg-ATP (5), and GTP (0.5).

Intracellular solution (μM) for current clamp: K-gluconate (135), NaCl (10), HEPES (10), MgCl_2_ (2), Mg-ATP (5), and GTP (0.5).

The pH of these solutions was adjusted to 7.3 with HCl. Osmolarity was adjusted to 305 mOsm by adding sucrose to the solution.

Drugs: 10 μM Tetraethylammonium chloride (TEA, T2265, Sigma-Aldrich) in the recording solutions was used to block the potassium current. 1–2 μM TTX (HY-12526, MCE) was used as an antagonist for transient sodium current, 2–3 μM riluzole (HY-B0211, MCE) for persistent sodium current, and 25 μM nimodipine (HY-B0265 MCE) for L-type calcium current. 15–20 μM 5-HT (H9523, Sigma-Aldrich) was used as an agonist for 5-HT receptors.

#### Liquid Junction Potential

The liquid conjunction potential was calculated as 10.4 mV with pH value adjusted to 7.3 by KOH, osmolarity adjusted to 310 mosM by sucrose, and the presence of 10 μM TEA in the recording solution. This value was not corrected in this study, in order to make our data comparable to those from our previous study of PICs (Dai and Jordan, 2010; Cheng et al., 2020).

#### Space-Clamp Issues

Incomplete space clamp is a problem for almost all studies using whole cell patch-clamp techniques (Dai and Jordan, 2011; Cheng et al., 2020). Any voltage-dependent current could be contaminated by the unclamped currents. Incidents of distortion of the inward currents by poor space clamp were observed in the present study. These included repetitive spikes (unclamped spikes) in voltage ramp, delayed inward currents (longer time to reach peak), bumps and notches in the inward currents. Recordings with any of these phenomena were excluded for calculation of PIC parameters in this study.

## Results

### Expression of PICs in 5-HT Neuron

The expression of PICs in 5-HT neurons of medulla has been reported in our recent study (Cheng et al., 2020). Consistent with that report, in this study we further showed that PICs were widely expressed in 5-HT neurons of medulla in ePet-EYFP mice. The electrophysiological data were collected from 258 5-HT neurons of P3–P6 ePet-EYFP mice in the present study. PICs were observed in 77% of recorded 5-HT neurons (198/258) in both PPR and MRN of medulla (Figure 1E top). Furthermore, 78% of PPR (107/137) and 75% of MRN 5-HT neurons (91/121) expressed the PICs, respectively (Figure 1E bottom). There was no significant difference between PPR (*n* = 107) and MRN (*n* = 91) 5-HT neurons in either onset (−47.4 ± 10 mV and −48.7 ± 7 mV) or amplitude (226.9 ± 138 pA and 259.2 ± 141 pA) of the PICs (Figure 1F). These parameters of PICs generally agree with recent report in brainstem 5-HT neurons of neonatal mice (Dai et al., 2016; Cheng et al., 2020).

### Four Patterns of PICs in 5-HT Neurons of Medulla

Based on our recording protocol (see section “Materials and Methods”), the PICs could be classified as an ascending PIC (a-PIC) evoked in the rising phase of the voltage bi-ramp and a descending PIC (d-PIC) generated in the falling phase of the bi-ramp. Although PIC has been described in this way in many studies, the ascending and descending PICs are actually an artificial description of the PICs.

The first pattern we described here was the PICs with a-PIC only (Figure 1G1, left). This pattern of PIC generated a counterclockwise hysteresis of PIC (Figure 1G1, right). The second pattern had both a-PIC and d-PIC with a-PIC amplitude larger than the d-PIC (Figure 1G2, left). This pattern exhibited a counterclockwise hysteresis (Figure 1G2, right). The third pattern had d-PIC only, leaving a passive response from the leak current in the rising phase of the voltage bi-ramp (Figure 1G3, left). This pattern generated a clockwise hysteresis of PIC (Figure 1G3, right). In contrast to the second pattern, the fourth pattern expressed both a-PIC and d-PIC with a-PIC smaller than the d-PIC (Figure 1G4, left). A clockwise hysteresis of PIC was shown in this pattern (Figure 1G4, right). Statistical results showed that 96% of the 5-HT neurons (191/198) displayed the counterclockwise hysteresis of PIC and 4% of the 5-HT neurons (7/198) showed the clockwise hysteresis of PIC.

### Multiple Components of PICs in 5-HT Neurons

PICs in mammals are primarily composed of three components: dihydropyridine (DHP) sensitive calcium component of PIC (Ca_PIC) (Zhang H. et al., 2006; Zhang M. et al., 2006; Fossat et al., 2010; Marcantoni et al., 2020), tetrodotoxin (TTX) sensitive sodium component of PIC (Na_PIC) and TTX- and DHP-resistance component (TDR_PIC) (Dai and Jordan, 2010, 2011; Cheng et al., 2020). Since there is no blocker specifically for TDR_PIC, in this study we defined the “Ca_PIC + TDR_PIC” as PICs recorded with TTX, “Na_PIC + TDR_PIC” as PICs with nimodipine, and TDR_PIC as PICs with TTX and nimodipine.

#### Na_PIC in 5-HT Neurons

**FIGURE 2:**
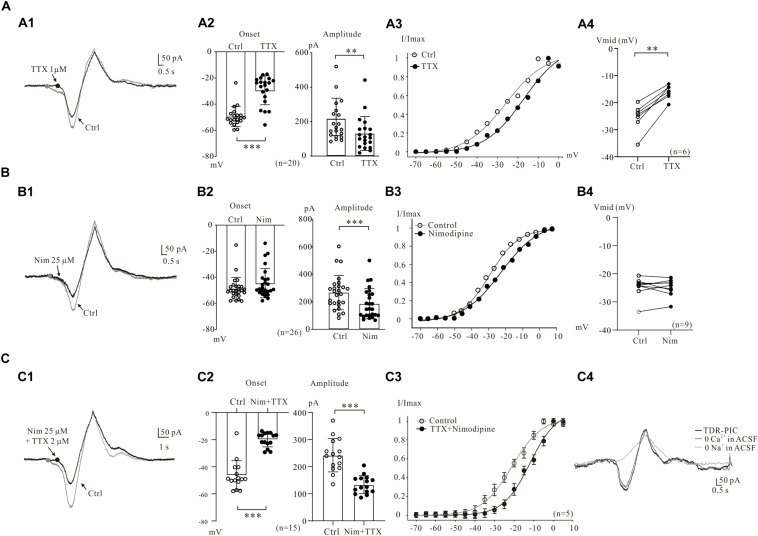
Multiple components of PICs. **(A)** Na_PIC was differentiated from PICs. **(A1)** Overlapped current traces recorded by voltage ramps in control (gray) and application of 2 μM TTX (black). **(A2)** Summary diagrams show the onset and amplitude of PICs recorded in control and presence of 1–2 μM TTX (*n* = 20). **(A3)** The half-maximal activations (V_mid_) of the PICs were calculated as −27.1 mV for control (open circles, **A2**) and −14.3 mV for TTX. **(A4)** Summary diagrams show the V_mid_ of PICs recorded in control and presence of 1–2 μM TTX (*n* = 6). **(B)** Ca_PIC was differentiated from PICs. **(B1)** Overlapped current traces recorded by voltage ramps in control (gray) and application of 25 μM Nimodipine (black). **(B2)** Summary diagrams show the onset and amplitude of PICs recorded in control and presence of 25 μM Nimodipine (*n* = 26). **(B3)** The V_mid_ were calculated as −27.9 mV for control (open circles) and −25.7 mV for Nimodipine. **(B4)** Summary diagrams show the V_mid_ of PICs recorded in control and presence of 25 μM Nimodipine (*n* = 9). **(C)** TDR_PIC was differentiated from PICs. **(C1)** Overlapped current traces recorded by voltage ramps in control (gray) and application of 1 μM TTX and 25 μM Nimodipine (black). **(C2)** Summary diagrams show the onset and amplitude of PICs recorded in control and presence of 1–2 μM TTX and 25 μM Nimodipine (*n* = 15). **(C3)** Results from five neurons, the V_mid_ were calculated for PICs (open circles) and TDR_PIC (*n* = 5). **(C4)** TDR_PIC was not changed after the calcium was removed from recording artificial cerebrospinal fluid (ACSF), but was completely blocked after complete removal of sodium from the recording solution. Error bars show SD. ^∗∗^*P* < 0.01, ^∗∗∗^*P* < 0.001, paired *t*-test.

The Na_PIC was examined by TTX (1–2 μM) in this study. Bath application of TTX (2 μM) substantially depolarized the onset of the PICs and decreased the amplitude of PICs (Figure 2A1). Results from 20 neurons showed that TTX (1-2 μM) significantly depolarized the onset by 20.1 ± 7 mV, from −49.5 ± 8 mV to −29.5 ± 10 mV (*P* < 0.001, *n* = 20, Figure 2A2 left) and reduced the amplitude of PICs by 25% from 224.2 ± 136 to 167.9 ± 98 pA (*P* < 0.01, *n* = 20, Figure 2A2 right). These results implicated that the Na_PIC accounted for 25% of the composite PIC.

Electrophysiological and modeling studies suggested that the onset of PICs was mainly determined by persistent Na^+^ channels, which may lead to a hyperpolarizing shift in their activation threshold (Cheng et al., 2020). In present study, we examined the effects of persistent Na^+^ channels on the onset of PICs by comparing the activation curves of PICs after blockade of persistent Na^+^ channels by TTX. An example is given in Figure 2A3, where the V_mid_ showed a dramatic depolarization, from −27.1 to −14.3 mV after bath administration of 2 μM TTX. Results from six 5-HT neurons showed that the V_mid_ was significantly depolarized by 8.4 ± 2 mV from −24.5 ± 5 mV to −16.1 ± 3 mV (*P* < 0.01, Figure 2A4).

#### Ca_PIC in 5-HT Neurons

The Ca_PIC was then examined by administration of 25 μM nimodipine in the present study. The nimodipine induced reduction of PICs amplitudes without changing the PICs onset in 5-HT neurons (Figure 2B1). Results from 26 neurons showed that nimodipine (25 μM) significantly reduced the amplitude of PIC by 30% from 267.7 ± 122 to 186.3 ± 108 pA (*n* = 26, *P* < 0.001, Figure 2B2, right). However, no significant change was found in onset of the PICs (from −48.8 ± 9 mV to −44.6 ± 11 mV, *n* = 26, *P* = 0.14, Figure 2B2, left). These results indicated that Ca_PIC accounted for 30% of the composite PIC.

Ca_PIC are considered to be the main components of PICs in many types of neurons (Hounsgaard and Mintz, 1988; Grueter et al., 2006), and study also demonstrated that Ca^2+^ channels blockers depolarized V_mid_ of PICs in spinal interneurons (Dai and Jordan, 2010). The effect of nimodipine on the V_mid_ of PICs was then investigated in the present study. A typical example is shown in Figure 2B3, where V_mid_ was depolarized by 3.8 mV from −26.9 to −23.1 mV with 25 μM nimodipine. Results from 9 neurons showed that no significant change was found in V_mid_ of the PICs with bath application of 25 μM nimodipine (control: −23.7 ± 6 mV; nimodipine: −24.9 ± 4 mV, *P* = 0.21; Figure 2B4).

#### TDR_PIC in 5-HT Neurons

Previous study indicated that TTX and DHP did not completely block the PICs and a new component of PIC, the TTX- and DHP-resistant PIC (TDR_PIC) was first described in spinal interneurons (Dai and Jordan, 2011). This novel PIC was shown to be mediated by sodium currents in spinal interneurons. In recent studies we also found the TDR_PIC in 5-HT neurons of brainstem (Cheng et al., 2019b; Dai and Cheng, 2019). In this study, we further investigated the TDR_PIC in medullar 5-HT neurons in the presence of 25 μM nimodipine and 2 μM TTX (Figure 2C1). Statistical results from 15 neurons indicated that nimodipine and TTX significantly depolarized the onset by 26.6 ± 6 mV from −46.3 ± 10 mV to −19.7 ± 4 mV (*n* = 15, *P* < 0.001; Figure 2C2, left) and reduced the amplitude by 45%, from 240.3 ± 60 pA to 132.6 ± 32 pA (*n* = 15, *P* < 0.001; Figure 2C2, right). These results confirmed that the TDR_PIC substantially accounted for a portion of the PICs in 5-HT neurons. These results also described the parameters of TDR_PIC in the medullar 5-HT neurons with onset of −19.7 ± 4 mV and amplitude of 132.6 ± 32 pA. Figure 2C3 illustrated the impact of the TTX and nimodipine on the V_mid_ of PICs. The kinetics of TDR_PIC determined by the Boltzmann function indicated that the half-maximal activation of TDR_PIC was −11.9 ± 2 mV (*n* = 5).

In this study we analyzed ionic component of the TDR_PIC by remove calcium and sodium ions from recording ACSF, respectively. Reducing calcium concentration to zero in the recording solution induced a little reduction of the amplitude of PICs (Figure 2C4). Further removal of sodium ions from the recording solutions completely blocked TDR_PIC. These results suggest that TDR_PIC in 5-HT neurons was mediated by sodium channels. This result generally agreed with previous report in spinal neurons (Dai and Jordan, 2011).

### Contribution of Multiple PICs to Excitability of 5-HT Neurons

Previous studies reported that PICs have an enhanced effect on neuronal excitability (Moritz et al., 2007; Heckman et al., 2008). Study in spinal motoneurons suggested that the relationship between motoneuron recruitment and decruitment of current thresholds were related to PICs activation; i.e., a hyperpolarization of the decruitment current threshold compared to the recruitment current threshold was a strong indicator of PICs activation and influenced on firing behavior (Bennett et al., 2001). In this study, we injected triangle current ramps into 5-HT neurons and measured the difference between the injected current at recruitment and decruitment (Figure 1D), i.e., ΔI, to infer the effect of putative PICs modulation on 5-HT neurons firing. To determine the contribution of multiple PICs to the excitability of 5-HT neurons, we recorded the 5-HT neurons in current-clamp mode with triangle current ramps (duration of 10 s, peak of 60–80 pA, and holding current of 0) and used concentrations of drugs similar to those used in voltage-clamp mode. This current protocol induced a slow depolarization and repolarization of membrane potential similar to those produced by voltage protocol for measurement of PICs (see section “Materials and Methods”).

**FIGURE 3:**
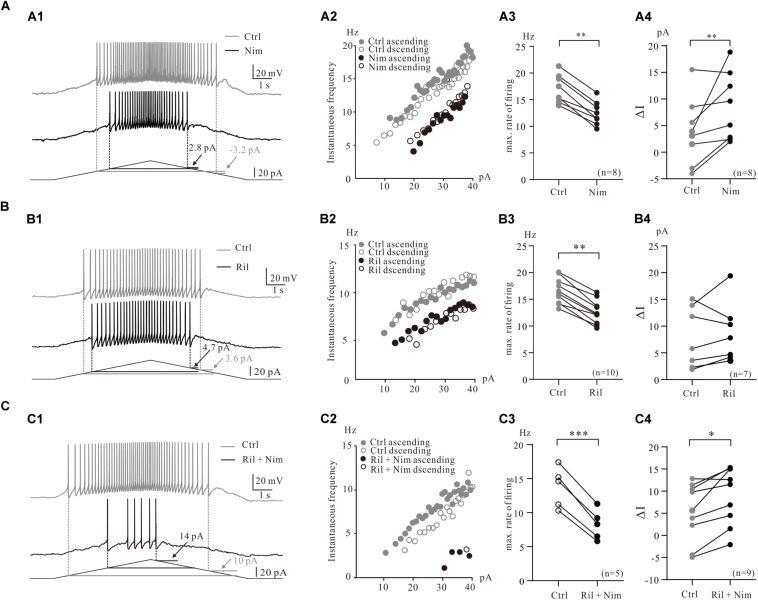
Contribution of multiple PICs to the firing properties of 5-HT neurons. **(A)** Contribution of Ca_PIC to the firing properties of 5-HT neurons. **(A1)** Overlapped voltage traces recorded by current ramp in control (gray) and application of 25 μM nimodipine (black). **(A2)** Instantaneous firing frequency/current relationship (iF-I relation) for both control and nimodipine condition. Depolarizing phase of the ramp represented by closed circles (control, gray closed circles; nimodipine, black closed circles) and repolarizing phase represented by open circles (control, gray open circles; nimodipine, black open circles). **(A3)** Graphs show the maximal frequency in control (gray circles) and in the presence of 25 μM nimodipine (black circles). **(A4)** Graphs show the ΔI in control (gray circles) and in the presence of 25 μM nimodipine (black circles). **(B)** The effects of Na_PIC on the firing properties of 5-HT neurons. **(B1)** Overlapped voltage traces recorded by current ramp in control and application of 2 μM Riluzole. **(B2)** Instantaneous firing frequency/current relationship for both control (gray circles) and Riluzole (black circles) condition. **(B3)** Graphs show the maximal frequency in control (gray circles) and in the presence of 2 μM Riluzole (black circles). **(B4)** Graphs show the ΔI in control (gray circles) and in the presence of 2 μM Riluzole (black circles). **(C)** Contribution of TDR_PIC to the firing properties. **(C1)** Overlapped voltage traces recorded by current ramp in control and application of 2 μM Riluzole and 25 μM nimodipine. **(C2)** Instantaneous firing frequency/current relationship for both control (gray circles) and Riluzole and Nimodipine (black circles) condition. **(C3)** Graphs show the maximal frequency in control (gray circles) and in the presence of 2 μM Riluzole and 25 μM nimodipine (black circles). **(B4)** Graphs show the ΔI in control (gray circles) and in the presence of 2 μM Riluzole and 25 μM nimodipine (black circles). Error bars show SD. ^∗^*P* < 0.05, ^∗∗^*P* < 0.01, ^∗∗∗^*P* < 0.001, paired *t*-test.

To examine the contribution of Ca_PIC to the firing properties of 5-HT neurons in medulla, we compared the instantaneous firing rate and ΔI of these neurons in control and in the presence of the Ca_PIC blocker nimodipine (Figure 3A1). Experimental results from a 5-HT neuron showed that nimodipine increased ΔI from −3.2 pA of control to 2.8 pA of nimodipine (Figure 3A1) and reduced the instantaneous firing rate with right-shift of the instantaneous firing frequency and ramp current relation (iF-I relation) (Figure 3A2). Statistical results from 8 5-HT neurons indicated that nimodipine significantly decreased the maximal firing rate by 4.6 ± 1 Hz from 17.0 ± 3 Hz to 12.4 ± 2 Hz (*n* = 8, *P* < 0.01, Figure 3A3) and increased the ΔI from 3.9 ± 6 pA to 8.5 ± 6 pA (*n* = 8, *P* < 0.01, Figure 3A4). These results suggested that Ca_PIC prolonged the discharge of 5-HT neurons, especially in the falling phase of the bi-ramp current.

Consistent with previous work that the riluzole reduced the excitability of 5-HT neurons in medulla (Cheng et al., 2020), experimental data from the present study indicated that riluzole substantially reduced the instantaneous firing rate of 5-HT neurons (Figures 3B1,B2). An example is given in Figure 3B1, where 2 μM riluzole increased the ΔI from 3.6 to 4.7 pA (Figure 3B1) and reduced the instantaneous firing rate of the 5-HT neuron with right-shifted of the iF-I relation (Figure 3B2). Statistical results showed that riluzole significantly decreased the maximal firing rate by 4.0 ± 2 Hz from 16.6 ± 2 Hz to 12.6 ± 2 Hz (*n* = 10, *P* < 0.01, Figure 3B3) and increased ΔI from 7.8 ± 5 pA to 8.8 ± 6 pA (*n* = 7, *P* = 0.41, Figure 3B4). The increment of ΔI was not statistically significant. These data suggested that blocked Na_PIC by riluzole decreased excitability of 5-HT neurons.

Next we investigated the contributions of TDR_PIC to regulation of repetitive firing of 5-HT neurons. 2 μM riluzole and 25 μM nimodipine clearly decreased the instantaneous firing rate and largely shifted the iF-I relation to the right (Figures 3C1,C2). As expected, the maximal firing rate significantly decreased by 5.4 ± 4 Hz from 13.7 ± 3 Hz to 8.2 ± 2 Hz (*n* = 5, *P* < 0.001, Figure 3C3) and the ΔI increased by 3.7 ± 5 pA from 5.2 ± 6 pA to 8.9 ± 6 pA (*n* = 9, *P* < 0.05, Figure 3C4). These results suggested that TDR_PIC contributed to regulation of excitability of 5-HT neurons.

### 5-HT Modulation of PICs

**FIGURE 4:**
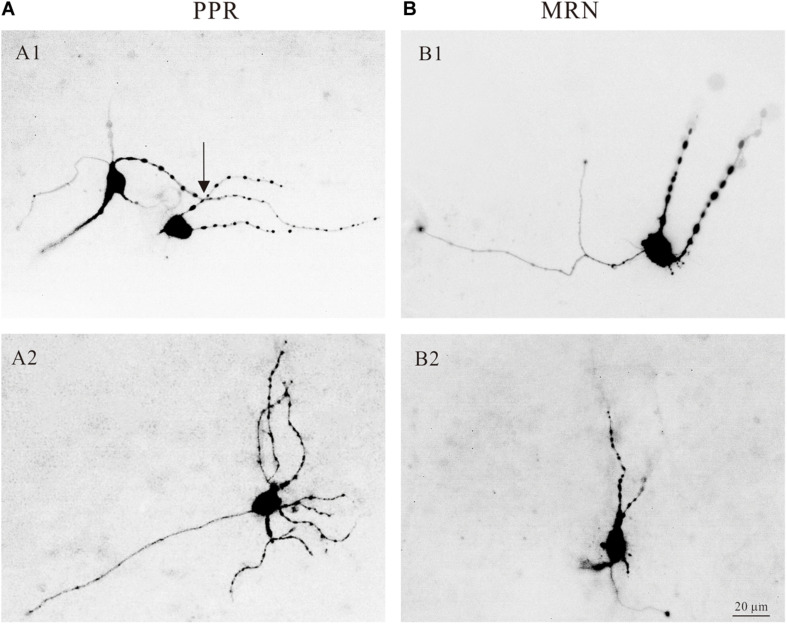
Morphology of serotonergic (5-HT) neurons. **(A1)** Two 5-HT neurons labeled with intracellular tetramethylrhodamine and located in PPR. The dendrites of the neurons with large spherical varicosities crossing the dendrites of the neurons (arrow). **(A2)** Tetramethylrhodamine filled a 5-HT neuron located in PPR with large spherical varicosities. **(B1,B2)** Two 5-HT neurons located in MRN with large spherical varicosities.

The central nervous system (CNS) of mammals is innervated by two morphologically distinct classes of 5-HT neural fibers: fine axons with minute varicosities and beaded axons characterized by large, spherical varicosities (Mamounas et al., 1991; Brown and Molliver, 2000). And the nerve fiber from the median raphe nucleus look relatively coarse with large spherical varicosities (Quentin et al., 2018). In the present study, we showed that most of the 5-HT neurons in the medulla had large varicosities. Four typical examples are shown in Figure 4, where 5-HT neurons located in both PPR (Figure 4A) and MRN (Figure 4B) areas and labeled with intracellular tetramethylrhodamine. Some 5-HT neurons had large varicosities intensively crossing their dendrites (Figure 4A1, arrow), anatomically supporting the potential serotonergic-modulation of 5-HT neurons in medulla (Quentin et al., 2018).

It might be surmised that the 5-HT neurons in medulla exert a widespread, diffuse influence in their nearby areas. However, the self-regulating effects of 5-HT neurons in medulla remain unclear. The effects of 5-HT on the PICs in spinal motoneurons and interneurons have been reported in many studies (Dai and Jordan, 2010; Revill et al., 2019). These studies demonstrated an enhancement of PICs by 5-HT. In this study, we further showed that activation of serotonergic receptors in 5-HT neurons enhanced PICs by hyperpolarizing PICs onset and/or increasing PICs amplitude.

**FIGURE 5:**
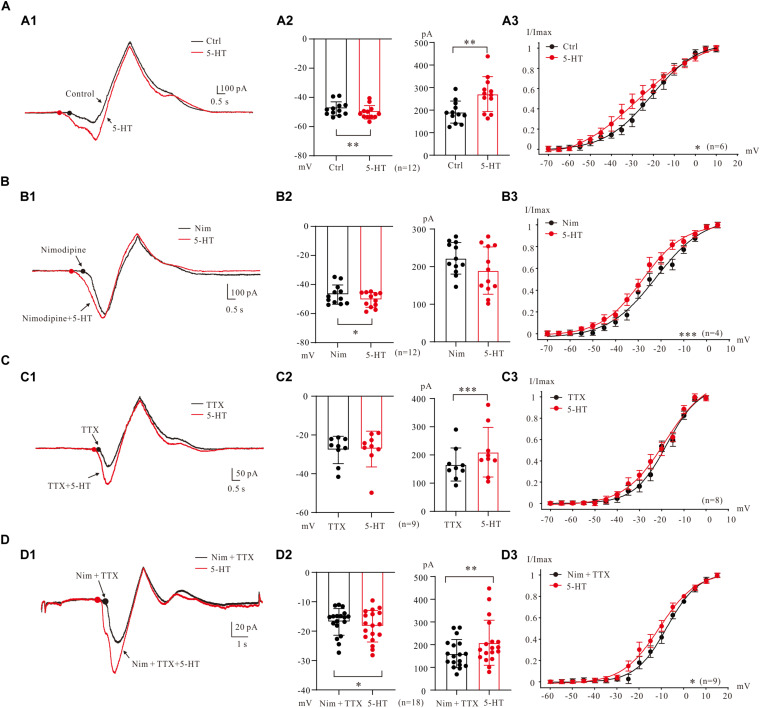
5-HT modulation of PICs. **(A)** The 5-HT modulation of PICs. **(A1)** Overlapped current traces recorded by voltage ramps in control (black) and 20 μM 5-HT (red). **(A2)** Summary diagrams show the onset and amplitude of PICs recorded in control and presence of 15–20 μM 5-HT (*n* = 12). **(A3)** 5-HT significantly hyperpolarized the V_mid_, *n* = 6, *P* < 0.05. **(B)** The 5-HT modulation of Na_PIC + TDR_PIC. **(B1)** Overlapped current traces recorded by voltage ramps in application of Nimodipine (25 μM, black) and 20 μM 5-HT (red). **(B2)** Summary diagrams show the onset and amplitude of Na_PIC + TDR_PIC recorded in control and presence of 15–20 μM 5-HT (*n* = 12). **(B3)** 5-HT significantly hyperpolarized the V_mid_, *P* < 0.001, *n* = 4. **(C)** The 5-HT modulation of Ca_PIC + TDR_PIC. **(C1)** Overlapped current traces recorded by voltage ramps in control (black) and 20 μM 5-HT (red). **(C2)** Summary diagrams show the onset and amplitude of Ca_PIC + TDR_PIC recorded in control and presence of 15–20 μM 5-HT (*n* = 9). **(C)** 5-HT did not significantly change the V_mid_, *n* = 8, *P* > 0.05. **(D)** The 5-HT modulation of TDR_PIC. **(D1)** Overlapped current traces recorded by voltage ramps in control (black) and 20 μM 5-HT (red). **(D2)** Summary diagrams show the onset and amplitude of TDR_PIC recorded in control and presence of 15–20 μM 5-HT (*n* = 18). **(D3)** 5-HT significantly hyperpolarized the V_mid_ of TDR_PIC, control: −6.7 ± 4 mV; 5-HT: −11.1 ± 5 mV, *P* < 0.05, *n* = 4. Error bars show SD. ^∗^*P* < 0.05, ^∗∗^*P* < 0.01, ^∗∗∗^*P* < 0.001, paired *t*-test.

In general, bath application of 5-HT (15–20 μM) increased PICs in 5-HT neurons through hyperpolarization of PIC onset and enhancement of PIC amplitude (Figure 5A1). Results from 12 neurons (Figure 5A2) showed that 5-HT hyperpolarized the onset by 2.5 ± 4 mV (from −47.8 ± 5 mV to −50.3 ± 4 mV, *P* < 0.01 Figure 5A2, left) and enhanced amplitude of PICs by 42% (from 191.1 ± 47 pA to 271.3 ± 74 pA, *P* < 0.01 Figure 5A2 right). 5-HT also induced hyperpolarization of V_mid_. Statistical results showed that 5-HT significantly hyperpolarized the V_mid_ by 6.7 ± 2 mV (control: −21.0 ± 3 mV; 5-HT: −27.7 ± 5 mV, *n* = 6, *P* < 0.05, Figure 5A3).

5-HT enhancement of Na_PIC + TDR_PIC (Harvey et al., 2006; Dai and Jordan, 2010) was also observed in 5-HT neurons of medulla (Figure 5B1). The Na_PIC + TDR_PIC was recorded with nimodipine (25 μM) presenting in the recording solution. 5-HT (15 μM) significantly hyperpolarized the onset by 3.6 ± 5 mV from −46.9 ± 6 mV to −50.5 ± 5 mV (Figure 5B2 left, *P* < 0.05, *n* = 12) with small decrease (<15%) in amplitude from 222.2 ± 40 pA to 189.5 ± 60 pA (Figure 5B2 right, *P* = 0.1, *n* = 12). 5-HT also substantially hyperpolarized the V_mid_ by 6.2 ± 4 mV (control: −22.1 ± 1 mV; 5-HT: −28.3 ± 3 mV, *P* < 0.001, *n* = 4, Figure 5B3). These results implicated that serotonergic modulation of Na_PIC + TDR_PIC in 5-HT neurons mainly targeted the voltage threshold for activation of the compound currents, i.e., the gating property of persistent sodium channels.

We then investigated effect of 5-HT on Ca_PIC + TDR_PIC. Experiment results showed that 5-HT dramatically increased the amplitude with little change in the onset (Figure 5C1). Analysis of 9 neurons showed that 5-HT increased amplitude by 26% from 166.2 ± 55 pA to 209.8 ± 83 pA (*P* < 0.001; Figure 5C2, right, *n* = 9) with no substantial change in onset (control: −27.7 ± 7 pA 5-HT: −27.3 ± 9 pA, *P* = 0.76; Figure 5C2, left, *n* = 9). 5-HT also induced a 0.7 ± 2 mV hyperpolarization of V_mid_, but this change was not significant (control: −17.7 ± 3 mV, 5-HT: −18.4 ± 3 mV, *n* = 8, *P* = 0.13; Figure 5C3). These results suggested that serotonergic modulation of Ca_PIC + TDR_PIC in 5-HT neurons mainly concentrated on the amplitude, i.e., the availability of channels mediating the compound currents.

Finally, we investigated the regulating effect of 5-HT on the TDR_PIC (Figure 5D1). Statistical results showed that 5-HT significantly hyperpolarized the onset by 1.5 ± 3 mV from −16.8 ± 4 mV to −18.3 ± 5 mV (*n* = 18, *P* < 0.05, Figure 5D2 left) and increased the amplitude by 30% from 160.7 ± 60 pA to 208.5 ± 97 pA (*n* = 18, *P* < 0.01, Figure 5D2 right). Further analysis showed that 5-HT induced a 4.4 ± 2 mV left-shift in half-maximal activation of TDR_PIC (from −6.7 ± 4 mV to −11.1 ± 5 mV, *P* < 0.05, *n* = 9, Figure 5D3). These results suggested that serotonergic modulation of TDR_PIC in 5-HT neurons targeted at both activation kinetics and availability of the sodium channels mediating the TDR_PIC.

### 5-HT Modulation of PICs in Current Clamp

**FIGURE 6:**
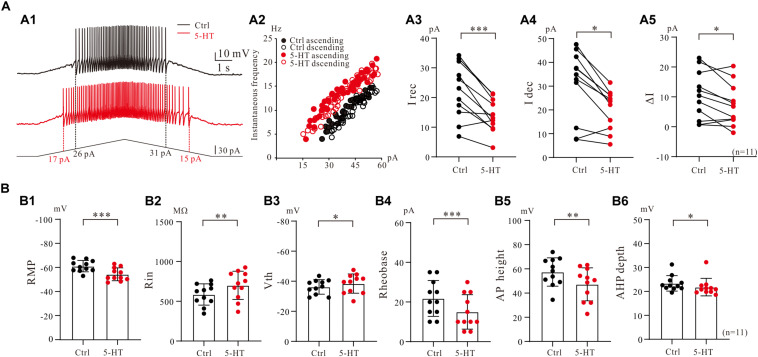
5-HT modulation of firing properties of 5-HT neurons. **(A1)** Repetitive firing evoked by bi-ramp current injected (10 s duration) into a 5-HT neuron (black). A 10 μM 5-HT was then applied to the recording solution (red). **(A2)** Instantaneous firing frequency/current relationship for both control and 5-HT conditions. **(A3–A5)** Values measured for recruitment current, decruitment current and ΔI in control (black) and bath application of 15 μM 5-HT (red). **(B1–B6)** Summary diagrams show 5-HT-induced significant changes in the membrane properties including resting membrane potential (RMP), input resistance (Rin), voltage threshold (Vth), rheobase, action potential height (AP height) and afterhyperpolarization depth (AHP depth) recorded in control and presence of 15 μM 5-HT (*n* = 11). Error bars show SD. ^∗^*P* < 0.05, ^∗∗^*P* < 0.01, ^∗∗∗^*P* < 0.001, paired *t*-test.

To assess serotonergic effect on neuronal excitability regulated by PICs, we recorded the 5-HT neurons in current-clamp protocol (Figure 6A1) and used the same concentrations of drugs as those used in voltage-clamp protocol for measurement of PICs. The experimental results showed that 5-HT increased the instantaneous firing rate with left-shift of iF-I relation. 5-HT also decreased recruitment current, decruitment current and recruitment difference ΔI. A typical example is shown in Figure 6A1, where 15 μM 5-HT reduced recruitment current by 9 pA (from 26 to 17 pA), decruitment current by 16 pA (from 31 to 15 pA) and the ΔI by 7 pA (from 5 to −2 pA). 5-HT also increased the instantaneous firing rate with left-shift of the iF-I relation (Figure 6A2). Analysis of 11 neurons showed that 5-HT significantly decreased the recruitment by 8.4 ± 5 pA (from 22.3 ± 9 pA to 13.9 ± 5 pA, *P* < 0.001, Figure 6A3), decruitment current by 10.3 ± 7 pA (from 30.7 ± 14 pA to 20.4 ± 8 pA, *P* < 0.01, Figure 6A4), and the ΔI by 3.3 ± 4 pA (from 10.5 ± 8 pA to 7.2 ± 7 pA, *P* < 0.05, Figure 6A5). These results indicated that 5-HT prolonged the discharge of 5-HT neurons induced by triangle current ramps, especially in the falling phase of the ramps. In addition to modulation of repetitive firing properties, 5-HT also potentially enhanced excitability of 5-HT neurons in terms of depolarization of membrane potential (Figure 6B1), increase of input resistance (Figure 6B2), hyperpolarization of voltage threshold (Figure 6B3), lowering of rheobase (Figure 6B4), reduction of action potential height (Figure 6B5), and reducing the AHP depth (Figure 6B6). Details of the data are summarized in Table 1.

**TABLE 1:** 5-HT effects on membrane properties of serotonin neurons.

	Control (*n* = 11)	5-HT	Changes
RMP, mV	−61.04	−54.25	6.82***
Rin, MΩ	585.4128	700.6170	115.358**
Vth, mV	−36.15	−38.36	−2.23*
Rheobase, pA	21.89	15.08	−6.84***
AP height, mV	57.411	47.313	−10.27**
AP half-width, ms	1.90.5	2.01	0.10.3
AHP depth, mV	23.43	21.84	−1.62*
AHP 1/2 decay, ms	247.261	234.560	−12.720

*Significant difference with p < 0.05; **p < 0.01; ***p < 0.001.

**FIGURE 7:**
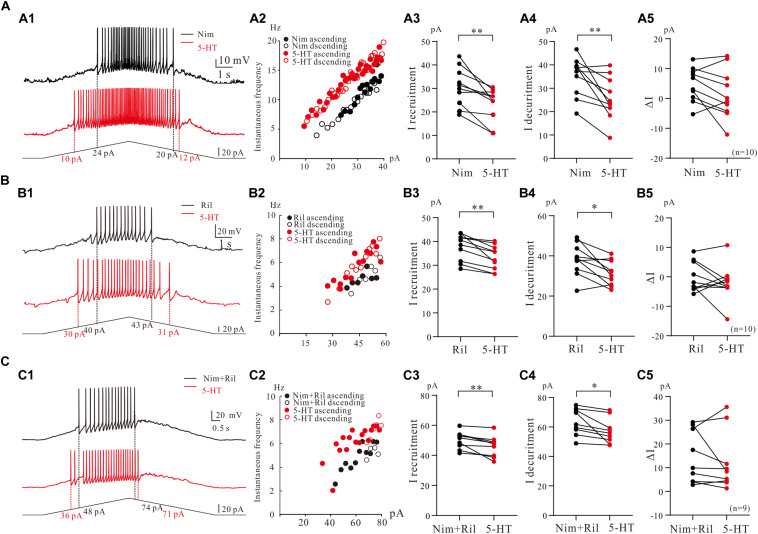
The effect of 5-HT on repetitive firing properties of 5-HT neurons with regulation of Na_PIC + TDR_PIC, Ca_PIC + TDR_PIC, and TDR_PIC. **(A1–C1)** Repetitive firing with regulation of Na_PIC + TDR_PIC, Ca_PIC + TDR_PIC, and TDR_PIC, respectively (black). Ten micrometers 5-HT was then applied to the recording solution (red). **(A2–C2)** Instantaneous firing frequency/current relationship recorded for control (black) and 5-HT (red). **(A3–C3)** Recruitment current measured for control (black) and 5-HT (red); **(A4–C4)** Decruitment current measured for control (black) and 5-HT (red). **(A5–C5)** ΔI calculated for control (black) and 5-HT (red). Error bars were shown as SD. ^∗^*P* < 0.05, ^∗∗^*P* < 0.01, paired *t*-test.

We then further investigated the effect of 5-HT on repetitive firing properties of 5-HT neurons with regulation of Na_PIC + TDR_PIC, Ca_PIC + TDR_PIC, and TDR_PIC. A typical example for Na_PIC + TDR_PIC-regulated repetitive firing was shown in Figure 7A1, where a 5-HT neuron was recorded in the presence of 25 μM nimodipine in the recording solution. 10 μM 5-HT increased the instantaneous firing rate and shifted the iF-I relation to the left (Figure 7A2). Statistic results showed that 5-HT significantly decreased the recruitment current by 6.3 ± 5 pA (from 30.6 ± 8 pA to 24.3 ± 6 pA, *P* < 0.01, *n* = 10, Figure 7A3), decruitment current by 9.5 ± 8 pA (from 35.3 ± 8 pA to 25.7 ± 9 pA, *P* < 0.01, *n* = 10, Figure 7A4) and ΔI by 3.2 ± 5 pA (control: 4.7 ± 5 pA, 5-HT: 1.4 ± 8 pA, *P* = 0.1, *n* = 10, Figure 7A5). There was no significant different in ΔI. These results suggested that 5-HT enhanced repetitive firing of 5-HT neurons by prolonging the Na_PIC + TDR_PIC-induced firing.

To study effect of 5-HT on repetitive firing regulated by Ca_PIC + TDR_PIC, riluzole (2–3 μM) was applied to the recording solution. An example was shown in Figure 7B1, where 10 μM 5-HT reduced recruitment current by 10 pA and decruitment current by 12 pA in this neuron (Figure 7B1). 10 μM 5-HT increased the instantaneous firing rate and slightly shifted the iF-I relation to the left (Figure 7B2). Results from 10 neurons showed that 5-HT induced a 4.3 ± 3 pA reduction of recruitment current (from 37.6 ± 5 pA to 33.3 ± 5 pA, *P* < 0.01, *n* = 10, Figure 7B3) and 6.2 ± 7 pA reduction of decruitment current (from 37.8 ± 7 pA to 31.6 ± 6 pA, *P* < 0.05, *n* = 10, Figure 7B4). Reduction of ΔI was observed in these neurons (control: 0.2 ± 5 pA, 5-HT: −1.7 ± 7 pA, *P* = 0.32, *n* = 10, Figure 7B5) but this reduction was not significant. Similar to the case of Na_PIC + TDR_PIC, these results implicated that 5-HT increased repetitive firing of 5-HT neurons by extending the Ca_PIC + TDR_PIC-regulated firing.

Finally, we investigated the effect of 5-HT on repetitive firing regulated by TDR_. TDR_PIC were recorded in the presence of 25 μM nimodipine and 2 μM riluzole in the recording solution (Figure 7C1), 5-HT (10 μM) increases the instantaneous firing rate and slightly shifted the iF-I relation to the left in this neuron (Figure 7C2). Statistical results from 9 neurons indicated that 5-HT significantly decreased recruitment current by 4.2 ± 3 pA (from 50.2 ± 5 pA to 46.0 ± 7 pA, *P* < 0.05, *n* = 9, Figure 7C3) and decruitment current by 6.2 ± 6 pA (from 63.5 ± 8 pA to 57.2 ± 8 pA, *P* < 0.05, *n* = 9, Figure 7C4). However, 5-HT reduced ΔI but did not substantially change ΔI (control: 13.3 ± 11 pA, 5-HT: 11.2 ± 12 pA, *P* = 0.45, *n* = 9, Figure 7C5). These results suggested that 5-HT could increase repetitive firing of 5-HT neurons by enhancing the TDR_PIC.

## Discussion

In the present study we systematically studied the PICs in 5-HT neurons of medulla in ePet-EYFP mice and characterized PICs on the basis of their electrophysiological and ionic properties. Using electrophysiological and pharmacological approaches, we demonstrated that PICs in 5-HT neurons consisted of Ca_PIC, Na_PIC, and TDR_PIC. We further explored the functional contribution of these three types of PICs to the firing properties of 5-HT neurons. More importantly, we studied serotonergic modulation of multiple PICs in 5-HT neurons. Our data showed that activation of 5-HT receptors in 5-HT neurons enhanced multiple components of the PICs, in terms of hyperpolarization of PIC onset and/or increase of PIC amplitude. This study suggested that 5-HT facilitated repetitive firing of 5-HT neurons by modulating multiple PICs.

### Multiple Components of PICs in Medullar 5-HT Neurons

PICs have been found in many types of neurons in vertebrate and invertebrate (Dai and Jordan, 2010; Hultborn et al., 2013; Ge et al., 2019; Revill et al., 2019). Previous studies suggested that PICs are composed of Na_PIC (Harvey et al., 2006; Zhong et al., 2007), Ca_PIC (Elbasiouny et al., 2006), and TDR_PIC (Dai and Jordan, 2011; Cheng et al., 2020). The Na_PIC activates at the lowest membrane potential, ranging between −70 to −50 mV (Hsu et al., 2018; Cheng et al., 2020), whereas the Ca_PIC activates at relatively higher membrane potentials within the range of −40 to −20 mV (Dai and Jordan, 2010), Previous study suggested that both Cav1.2 and Cav1.3 calcium channels activated more hyperpolarized potentials, about −60 mV (Lipscombe et al., 2004). Therefore, we speculate that the Ca_PIC in 5-HT neurons may not be dominated by Cav1.3. Study by Dai and Jordan reported that the TDR_PIC in spinal neurons activates between −20 and −30 mV, higher than Na_PIC and Ca_PIC (Dai and Jordan, 2011). Although PICs in 5-HT neurons in brainstem have been studied recently in our studies (Cheng et al., 2020; Ge and Dai, 2020), detail of PICs in 5-HT neurons of medulla, especially their modulatory properties by serotonin are still missing.

In general, the properties of PICs in 5-HT neurons are consistent with those in spinal neurons in terms of electrophysiological and pharmacological properties. However, the amplitude of PICs are quite different between the 5-HT neurons and spinal motoneurons. In spinal motoneurons, PIC amplitude is 1–2 times the rheobase (Harvey et al., 2006; Dai and Jordan, 2010). In medullar 5-HT neurons, however, PIC amplitude is 10–12 times the rheobase (Figure 1F and Table 1). Similar results are also observed in 5-HT neurons of dorsal raphe nucleus in juvenile mice, where PIC amplitude is about nine times the rheobase (Ge and Dai, 2020). Therefore, we speculate that these differences may be due to different types of neurons and functional roles in regulating neuronal excitabilities for different behaviors such as mental (Meera et al., 2003; Kang et al., 2019) or physiological behaviors (Fernandez-Fernandez et al., 2018; Hsu et al., 2018).

### Contribution of Multiple PICs to Excitability of 5-HT Neurons

PICs are long-lasting, voltage-dependent currents that can amplify synaptic inputs and generate plateau potentials in spinal neurons (Svirskis and Hounsgaard, 1997; Heckman et al., 2003, 2008). Brainstem-derived neuromodulatory inputs produce dendritic PICs that control the state of excitability of the motoneuron (Heckmann et al., 2005). PICs are functionally powerful to promote self-sustained firing of motoneurons (Elbasiouny et al., 2010). The PICs promoting the excitability of neurons in adult cats (Hounsgaard and Kiehn, 1985), mice (Carlin et al., 2000; Dougherty et al., 2008; Bellardita et al., 2017), rats (Li and Bennett, 2003), turtles (Hounsgaard and Mintz, 1988), and frogs (Perrier and Tresch, 2005) are predominantly mediated by L-type calcium channels or to some extent by Na_PIC. In this study we demonstrated that blocking Ca_PIC or Na_PIC reduced the maximum rate of repetitive firing and shifted the iF-I relation to the right (Figures 3A,B). These results were consistent with the data from voltage clamp recordings in which TTX and/or nimodipine reduced composite PICs and shifted (depolarized) the activation voltage to the right (Figures 2A,B). Furthermore, our data showed that 96% of the 5-HT neurons (191/198) displayed the counterclockwise hysteresis of PIC (a-PIC > d-PIC) in voltage ramps (Figure 1G), and 83.3%, of 5-HT neurons exhibited ΔI > 0 in the recording conditions of current ramps (Figures 3A4,B4,C4, control condition, *n* = 24). These results suggest that the hysteresis of PIC is related to the discharge of neurons (Powers and Heckman, 2015, 2017). However, there is no noticeable difference in the gain of the F-I relation of the ascending and descending current ramps (Figure 6). This result was different from previous observations in spinal motoneurons (Bennett et al., 2001; Venberk and Kalmar, 2014). In addition, adding riluzole or nimodipine caused a right-shift of the F-I relation with no significant change in the gain (Figures 3, 7). The present study suggests that PIC has a relatively small effect on repetitive firing of 5-HT neurons of medulla. Furthermore, our data showed that the proportion of Na_PIC and Ca_PIC in 5-HT neurons was about 25 and 30% less than the composite PIC, respectively, suggesting that the TDR_PIC might play a significant role in the discharge of 5-HT neurons.

TDR_PIC was first discovered in rodent spinal neurons (Dai and Jordan, 2011). The same current was also found in 5-HT neurons of the brainstem in our recent study (Cheng et al., 2020). This is a high voltage-activated sodium current and generally thought to have little effect on the excitability of neurons. Unexpectedly, however, this current exhibited an important effect on repetitive firing of 5-HT neurons. In this study we first confirmed that TDR_PIC in the 5-HT neurons was mediated by sodium currents (Figure 2C4), the same as we reported in spinal neurons (Dai and Jordan, 2011) and characterized this current with biophysical properties (onset −19.7 ± 4 mV, amplitude 132.6 ± 32 pA, V_mid_ −11.9 ± 2 mV; Figures 2C2,C3). We found that the maximum discharge rate of 5-HT neurons dropped dramatically from composite PICs-mediated repetitive firing to TDR_PIC-mediated firing (Figure 3C3), consistent with substantial reduction of composite PICs to TDR_PIC after bath application of TTX and nimodipine (Figure 2C1). Our data further showed that the TDR_PIC-induced difference between the recruitment and decruitment ΔI was generally bigger than zero (Figure 3C4), suggesting that TDR_PIC did not induce prolonged repetitive firing in falling phase of bi-ramp currents with respect to the rising phase. Our study suggested that TDR_PIC was an unique sodium current which contributed to maintenance of repetitive firing of 5-HT neurons at higher voltage range.

### 5-HT Modulation of Multiple PICs and Neuronal Excitability

As reported in spinal motoneurons PICs potentiated by neuromodulators enhanced motoneuron excitability (Hounsgaard and Kiehn, 1989; Lee and Heckman, 2000). Numerous studies suggested that descending serotoninergic input from the medulla to spinal cord is primary modulating system for regulation of excitability of interneurons and motoneurons in spinal cord. 5-HT neurons of medulla play a critical role in initiating and maintaining locomotion (Schmidt and Jordan, 2000). In this study we explored the serotonergic modulation of excitability of serotonergic neurons in medulla. Our data clearly showed that 5-HT substantially increased excitability of 5-HT neurons in terms of depolarization of resting membrane potential, increase of input resistance, reduction of afterhyperpolarization, and hyperpolarization of voltage threshold for action potential generation (Table 1 and Figure 6B). These results were consistent with previous studies in 5-HT-modulated membrane properties of spinal neurons (Zhong et al., 2006; Dai et al., 2009), suggesting that the serotonergic modulation of serotonergic neurons in medulla may not be different from serotonergic modulation of spinal neurons in terms of regulation of membrane properties. The modulatory difference between medullar and spinal neurons should be shown in their different functional roles.

5-HT modulation of PICs has been studied intensively in spinal and brainstem neurons for Ca_PIC (Hounsgaard and Kiehn, 1989; Dai and Jordan, 2010) and Na_PIC (Hsiao et al., 1998). Enhancement of PICs was generally observed in these studies, and consistent with these results, we showed that 5-HT enhanced PICs by hyperpolarizing PIC onset and amplifying PIC amplitude in 5-HT neurons (Figure 5A). Furthermore, 5-HT modulation of multiple PICs was mainly shown as hyperpolarization of onset for Na_PIC + TDR_PIC (Figure 5B), enhancement of amplitude for Ca_PIC + TDR_PIC (Figure 5C), and both lowering of onset and increase of amplitude for TDR_PIC (Figure 5D). These results suggested that 5-HT modulation of PIC could be mediated by regulating gating property (voltage dependency) for Na_PIC, enhancing availability of channel conductance for Ca_PIC, and modulating both gating kinetics and channel availability for TDR_PIC, respectively. These results demonstrated unique property of serotonergic modulation of PICs in 5-HT neurons and unveiled different mechanisms underlying the modulation. However, compared with serotonergic regulation of neuronal excitability with altering membrane properties as shown in Table 1, the same modulation of PICs appeared to have relatively smaller effect on the excitability of 5-HT neurons. Therefore, we suggest that serotonergic modulation of PICs only partially contributed to enhancement of excitability of 5-HT neurons in medulla.

Agreeing with the above results, our data further demonstrated that 5-HT also enhanced the excitability of 5-HT neurons by modulating multiple PICs in 5-HT neurons (Figure 7). This enhancement was shown as left-shift of the iF-I relation and reduction of the recruitment and decruitment currents through modulation of composite PICs (Figure 6A), Na_PIC + TDR_PIC (Figure 7A), Ca_PIC + TDR_PIC (Figure 7B), and TDR_PIC (Figure 7C). In fact, it was hyperpolarization of onset and/or increase of amplitude of the PICs that underlay the enhancement of excitability of 5-HT neurons. The notable result was the 5-HT enhancement of TDR_PIC which facilitated the repetitive firing of the 5-HT neurons. The TDR_PIC was a high voltage-activated sodium current which accounted for 55% of the composite PICs. Its functional role has never been studied previously since it was first reported in spinal interneurons (Dai and Jordan, 2011). For the first time we demonstrated that TDR_PIC contributed to regulation of neuronal excitability which could be amplified by 5-HT.

### Serotonergic Modulation of 5-HT Neurons in Medulla

5-HT neurons originate from a cluster of nuclei located in the pons and medulla of the brainstem (Dahlström, 1964). The rostral groups (B4-B9) generally project to the forebrain; and the caudal groups in medulla (B1-B3) project terminals to the spinal cord (Adams-Ray et al., 1964). The serotonergic system forms a diffuse network within the CNS and plays a significant role in the regulation of mood and cognition (Quentin et al., 2018; Vadodaria et al., 2018). 5-HT neurons of medulla play an essential role in generating locomotion (Liu and Jordan, 2005). In dorsal raphe neurons, the release of 5-HT from varicosities in dendrites and/or axonal varicosities is independent of classical synapses and can be induced by membrane potential depolarization (Quentin et al., 2018). In this study, we found the structure of varicosities on dendrites of 5-HT neurons of medulla (Figure 4). Morphological analysis indicated that these dense spherical varicosities intensively crossed 5-HT neurons, anatomically supporting serotonergic modulation of 5-HT neurons in medulla. The morphological data (Figure 4) also supported our electrophysiological results that 5-HT increased the excitability of 5-HT neurons through serotonergic modulation of multiple PICs in 5-HT neurons. Previous studies reported that the reuptake of 5-HT is mediated primarily by serotonin reuptake transporter (SERT), and SERT is located on soma and dendrites of 5-HT neurons rather than within the presynaptic area. Inhibition of the SERT is a conventional method for clinical treatment of depression (Mohammad-Zadeh et al., 2008; Szoke-Kovacs et al., 2020). These results implicated putative self-regulatory loop of serotonergic system in brainstem. However, this issue was not investigated in the present study.

In many previous studies (Martin-ruiz and Ugedo, 2001; Diaz et al., 2012), it has been confirmed that 5-HT1A and 5-HT2B receptors present in high density on serotonergic cell body areas, in particular the dorsal raphe neurons. 5-HT1A receptors function as somatodendritic autoreceptors, involved in the negative feedback modulation of serotonergic neuronal activity (Riad et al., 2000; Quentin et al., 2018). And 5-HT1A receptors are coupled to the opening of potassium channels (Gadgaard and Jensen, 2020). In the present study, our data showed that 5-HT reduced the amplitude of the Na_PIC + TDR_PIC (Figure 5B2 right), so we speculate that this reduction could be mediated by the 5-HT1A receptors. Some other studies demonstrated that stimulation of 5-HT2B receptors in dorsal raphe increased the excitability of 5-HT neurons and extracellular serotonin, supporting an excitatory effect of this receptor on serotonergic neuron activity (Doly et al., 2008). However, there is few report about the distribution of other subtypes of 5-HT receptors on 5-HT neurons of medulla (Quentin et al., 2018). On the other hand, however, 5-HT neurons in brainstem have been shown to play essential role in initiating locomotion in rodents (Liu and Jordan, 2005), And 5-HT7 receptors are required for the production and coordination of 5-HT-induced locomotor-like activity in the neonatal mouse and are important for the coordination of voluntary locomotion in adult mice (Liu et al., 2009). Based on these results, we could expect that serotonergic modulation of 5-HT neurons in medulla could be mediated through activation of 5-HT1A and 5-HT2B receptors for regulating neuronal excitability and activation of 5-HT7 receptors for generation of locomotion. A further study is required to investigate this issue.

## Conclusion

PICs were composed of Na_PIC, Ca_PIC and TDR_PIC in 5-HT neurons of medulla. 5-HT enhanced the multiple PICs in terms of hyperpolarization of onset and/or increase of amplitude. 5-HT upregulated the excitability of 5-HT neurons through serotonergic modulation of PICs in 5-HT neurons.

## Data Availability Statement

All datasets generated for this study are included in the article/supplementary material, further inquiries can be directed to the corresponding author/s.

## Ethics Statement

The animal study was reviewed and approved by the East China Normal University Laboratory Animal Center.

## Author Contributions

YD and YC conceived and designed the research, wrote, and revised manuscript. YC and NS performed the experiments. YC and RG analyzed the data. YD, YC, NS, and RG approved the final version of the manuscript. All authors contributed to the article and approved the submitted version.

## Conflict of Interest

The authors declare that the research was conducted in the absence of any commercial or financial relationships that could be construed as a potential conflict of interest.
